# The stereotype content model and mental disorders: Distinct perceptions of warmth and competence

**DOI:** 10.3389/fpsyg.2023.1069226

**Published:** 2023-02-14

**Authors:** Ramona C. Allstadt Torras, Corinna Scheel, Angela R. Dorrough

**Affiliations:** ^1^Social Cognition Center Cologne, University of Cologne, Cologne, Germany; ^2^Developmental Psychology and Clinical Psychology of the Lifespan, Department of Psychology, University of Siegen, Siegen, Germany

**Keywords:** stereotypes, stereotype content model, SCM, warmth, competence, mental disorders, mental illness

## Abstract

This work investigates the perception of eight different mental disorders within the Stereotype Content Model (SCM). The presented study (*N* = 297) includes a sample representative for the German population in terms of age and gender. Results reveal distinct warmth and competence evaluations for people with different mental disorders, e.g., people with alcohol dependence were seen as less warm and less competent than people with depression or phobia. Future directions and practical implications are discussed.

## 1. Introduction

More than one third of the northeastern German population is affected by mental disorders (12-month prevalence of 36.3%; Asselmann et al., 2019), which are associated with far reaching restrictions on daily life such as negative impacts on personal well-being, social life, and work productivity as well as a significant decreased quality of life and increased impairment (Alonso et al., 2004). In addition to these direct negative impacts, people with mental disorders are often stigmatized by their environment. Angermeyer et al. (2013) showed that the acceptance of treatment offered by mental health professionals has increased in the period from 1990 to 2011 in Germany. However, public attitudes (which were conceptualized as a more or less positive emotional reaction and the desire for social distance) toward people with major depression and alcohol dependency did not increase significantly and attitudes toward people with schizophrenia have even worsened. Similar studies conducted in the US (e.g., Pescosolido et al., 2010), Australia (Reavley and Jorm, 2011), Austria (Grausgruber et al., 2009), England, and Scotland (Mehta et al., 2009) also failed to find a decrease in stigmatization toward people suffering from mental disorders (for a meta-analysis, see Schomerus et al., 2012). Schmitt et al. (2014) demonstrated in two meta-analyzes that perceived discrimination has subsequent negative effects on several aspects of psychological well-being and even hinders people with mental health problems from seeking help (Clement et al., 2015; Henderson et al., 2017), thus impeding recovery. Stigmatization can also lead to additional restrictions for people with mental disorders with regard to obtaining or maintaining employment in accordance with their education or abilities (Corrigan, 2004). Furthermore, research indicates that people with mental disorders experience work-related discrimination (Yoshimura et al., 2018) and are systematically disadvantaged in comparison to people with physical disorders in the labor market (Hipes et al., 2016). Although the causes of discrimination are manifold, generalized beliefs regarding a group of people based on their group membership (i.e., stereotypes) are seen as one cognitive component (e.g., Ashmore and Del Boca, 1981; Fiske, 1999; Kanahara, 2006). Stereotypes are often automatically activated when encountering a person belonging to a specific group (e.g., Macrae and Bodenhausen, 2000) and can subsequently shape behavior toward that person (Cuddy et al., 2007). Because of the wide-ranging negative consequences resulting from stereotypes, especially for people with mental health problems, understanding and counteracting stereotypes toward this minority is crucial.

According to the Stereotype Content Model (SCM), which has been validated in a wide range of cultures (Cuddy et al., 2009; Durante et al., 2013, 2017; Fiske and Durante, 2016), stereotypes about a social group follow two fundamental dimensions of social judgment, namely warmth and competence (Fiske et al., 2002; Cuddy et al., 2008). Research has shown that the social perception of various groups (such as people with different nationalities, professions, social backgrounds, and religions, and so on; for a summary see Fiske, 2018) varies along these two dimensions. Apart from different social and cultural groups, some studies have investigated stereotypes and stigmas for people with mental disorders. Most of this research focused on mental disorders in general (Phelan et al., 2000; Corrigan, 2004; Hinshaw and Stier, 2008; Mehta et al., 2009; Asbrock, 2010) or investigated only one or a small number of specific categories of mental disorders such as depression (Griffiths et al., 2008), anxiety (Curcio and Corboy, 2020), and schizophrenia (e.g., Link et al., 2004; Rüsch et al., 2005). A study including the general category “mentally ill“, found that participants attribute a moderate value on the warmth dimension and almost no competence to this group (Cuddy et al., 2009). Asbrock (2010) shows that people with mental and physical disorders are both located in the same cluster together with social categories such as people living in homelessness, welfare recipients, and people receiving unemployment. People with mental disorders are perceived to be lower in warmth and competence than people with physical disorders. Angermeyer and Dietrich (2006) criticized the use of broad categories or selected disorders, as this may possibly lead to incorrect conclusions by neglecting the diverse nature of different mental disorders. Furthermore, the perception of mental disorders has seldom been systematically investigated within the scope of the most prominent model of stereotype perception, the SCM: Fiske (2012) reported that mental health issues such as eating disorders, depression, and autism were located in the left lower corner (i.e., neither warm nor competent) of the coordinate system spanning the two dimensions. Sadler et al. (2012) found that, analogous to Cuddy et al. (2009), the overarching category of mentally ill people was perceived to be moderate in warmth and relatively low in competence. Furthermore, specific stereotypes about different disorders revealed four distinct clusters distributed across the two-dimensional coordinate system: (1) low warmth/low competence (e.g., addictions, and schizophrenia), (2) moderate warmth/moderate competence (e.g., anxiety, depression, eating disorders, and obsessive–compulsive disorder), (3) low warmth/moderate competence (sociopathy and violent criminals), (4) high warmth/low competence (e.g., neurocognitive disorders and mental retardation). Sönmez and Karaoğlu (2022) had a variety of mental disorders rated on the dimensions of the SCM by Turkish psychology students. Boysen (2017) used the SCM to measure the perception of 17 different mental disorders and focused on aggregated perceptions of typically masculine and feminine disorders and Gärtner et al. (2022) have shown that the SCM is also applicable to the self-stigmatization of people with various mental disorders.

The studies described above that have assessed the public perception of several mental disorders on the warmth and competence dimensions (Sadler et al., 2012; Boysen, 2017) included random samples from the Mechanical Turk panel provider in the United States of America or a sample of Turkish undergraduates in psychology (Sönmez and Karaoğlu, 2022). However, the role of the respondents’ demographic characteristics on stereotypical perceptions of people with mental disorders has not been fully determined. According to a review article by Angermeyer and Dietrich (2006) 11 studies reported more negative attitudes of male compared to female participants toward people with mental disorders, whereas 6 studies showed opposite results. In addition, 18 studies did not report any effects of participant gender on how different mental disorders were perceived. Furthermore, in 32 out of 33 studies they found a positive association between participants’ age and the stereotypical perception. Holzinger et al. (2012) summarized that emotional reactions toward people with mental disorders differed between the genders in that women expressed more positive reactions and less anger, but showed more anxiety than men. A nation-wide cross-sectional survey on mental health literacy in Singapore with 3,006 participants revealed that attitudes towards people with mental disorders were more negative amongst older participants and participants with male gender (Yuan et al., 2016). In sum, the results regarding the impact of age and gender on the stereotype perception of people with mental disorders are mixed. However, since the previous findings also do not indicate that these variables are irrelevant in this context, we select a sample that is representative of the German population in terms of age and gender. With regard to cultural differences, Cuddy et al. (2009) indicated with their studies that there can be similarities but also differences with regard to how various social categories are perceived on the warmth and competence dimensions. Therefore, we consider it reasonable to extend the existing data of mapped stereotypes towards people with specific mental disorders with a sample from Germany.

The present study shall provide a general picture of warmth and competence stereotypes for different mental disorders in Germany. In order to address the most relevant and prominent mental disorders, we chose eight mental disorders from the ICD-10 (World Health Organization [WHO], 1993) and included highly prevalent affective and anxiety disorders (major depressive disorder (MDD; F32), specific phobia (F40.29), obsessive compulsive disorder (OCD; F42)), one personality disorder, additional societally relevant disorders [emotional-unstable personality disorder, borderline type (BPD; F60.31), alcohol dependence disorder (ADD; F10.2); anorexia nervosa (F50.0)], and also other less prevalent but commonly known disorders [schizophrenia (F20), pathological stealing/kleptomania (F63.2)]. The remaining chapter V disorders about organic (including symptomatic) mental disorders (F0), mental retardation (F7), and disorders of psychological development (F8) as well as behavioral and emotional disorders with onset typically occurring in childhood and adolescence (F9) describe mental disorders in a broader sense and were therefore not included.

The materials and data can be found at https://osf.io/ukzn2/. We did not pre-register the hypotheses and did not estimate power beforehand, as the data was collected in 2016 and both practices had not yet been added to our research repertoire at that time. A post-hoc sensitivity analysis assuming Chi square test as our main test using G*Power (Faul et al., 2007) indicated that we can detect small to medium effects (Cohen’s *ω* =0.22; *α* = 0.05, *χ*^2^-test) with the available sample and a power of 1−*β* = 0.80.

## 2. Hypotheses

*The main hypothesis predicts distinct patterns for different mental disorders in terms of warmth and competence (H1)*. The following disorder-specific hypotheses are based on previous research concerning stereotypes about different mental disorders.

### 2.1. Schizophrenia

In studies on social perception, participants stated a high desire to maintain social distance from people with schizophrenia and associated such individuals with dangerousness (e.g., Link et al., 1999; Thompson et al., 2002; Read et al., 2006; Ahmed et al., 2020), rejection (Angermeyer and Matschinger, 2004, 2005), anger (Angermeyer and Matschinger, 2003; Ahmed et al., 2020), and perceived dependency (Angermeyer and Matschinger, 2003) as well as fear and unpredictability (Levey et al., 1995; Angermeyer and Matschinger, 2003; Read et al., 2006). These findings suggest that within the SCM, people with schizophrenia might be perceived to be low in warmth. Pescosolido et al. (1999) found that only 25.7% of a representative sample rated people with schizophrenia as very or somewhat able to manage treatment decisions, which might result in low ratings of competence. In line with the results by Sadler et al. (2012), who located this group in the low warmth/low competence cluster, *we hypothesize that people with schizophrenia are perceived to be low in warmth and competence within the SCM (H2a)*.

### 2.2. Alcohol dependence disorder

Schomerus et al. (2012) concluded that people with ADD are perceived to be equally or more dangerous and more unpredictable than people with other mental disorders; in addition, participants reported that their desire for social distance toward this group is even greater. Furthermore, people with ADD are seen to be much more responsible for their own disorder as compared to non-substance abuse disorders. Both the high degree of perceived dangerousness and unpredictability as well as the ascribed responsibility led to a desire for social distance and a low level of sympathy (Feldman and Crandall, 2007), which might be associated with low warmth ratings within the SCM. In Pescosolido et al. (1999), 35.5% of participants rated individuals with drug dependence as “not at all” able to make adequate decisions, suggesting that affected people might be perceived as low in competence. In line with the described findings and the results by Sadler et al. (2012), who located this group in the low warmth/low competence cluster, *we hypothesize that people with ADD will be rated as low in warmth and competence within the SCM (H2b)*.

### 2.3. Major depression disorder

Pescosolido et al. (1999) showed that almost two-thirds of their participants rated people with MDD as being able to make proper medical treatment decisions. Thus, moderate to low competence ratings in the SCM are possible. Regarding the warmth dimension, a study by Link et al. (1999) reveals that, in comparison to other disorders, people suffering from MDD are perceived to be less likely to become violent. In Sadler et al. (2012), people with MDD fell into the “internal” cluster (moderate warmth *M* = 2.86/moderate competence *M* = 3.08). *We, therefore, assume that people with MDD are perceived to be moderate in warmth and competence (H2c)*.

### 2.4. Borderline personality disorder

Several studies indicate that healthcare professionals, in particular, have negative attitudes toward people with BPD. They predominantly perceive them to be manipulative and difficult and report that they make them angry or annoyed (Deans and Meocevic, 2006; Ross and Goldner, 2009). This suggests that people with BPD may also be perceived to be low in warmth by the general public. The literature provides only little evidence regarding the perceived competence of people with BPD, *which leads us to the hypothesis that they are perceived to be low in warmth and moderate in competence (H2d)*.

### 2.5. Anorexia nervosa

People with eating disorders (i.e., bulimia and anorexia) were rated as being more fragile than people with MDD (Roehrig and McLean, 2010). As for ADD, people with eating disorders, including anorexia, are perceived to be responsible for their own condition (Feldman and Crandall, 2007; Crisafulli et al., 2008; Roehrig and McLean, 2010; Ebneter et al., 2011). This potentially leads to a desire for social distance and a low level of sympathy and, thus, social perception ratings of low warmth. According to Sadler et al. (2012), eating disorders are part of the moderate warmth/moderate competence cluster (“internal cluster” in Sadler et al., 2012). *Thus, we hypothesize that people with anorexia nervosa are perceived as being low in warmth and moderate in competence (H2e)*.

### 2.6. Specific phobia

Little has been published on the stereotypical perception of people with phobias to date. In Sadler et al. (2012), people with anxiety (in general) were located in the medium warmth/medium competence cluster. Angermeyer and Dietrich (2006) showed that negative attitudes toward people suffering from anxiety disorders (in general) are not as pronounced as toward disorders such as ADD and schizophrenia. This appears consistent with the tendency of people with anxiety and phobic disorders to generally show more avoidance tendencies and inwardly directed coping rather than, for example, outwardly aggressive behavior. This would suggest no danger for other people and therefore comparably high warmth ratings. Concerning competence, there are no findings that would suggest high or low ratings of competence. *We therefore hypothesize that people with phobic disorders are perceived to be high in warmth and moderate in competence (H2f)*.

### 2.7. Obsessive–compulsive disorder

Sadler et al. (2012) found that people with OCD are perceived to be moderate in warmth and competence. As we have no additional information on social judgments of people with OCD, *we hypothesize that they are perceived to be moderate in warmth and competence within the SCM (H2g)*.

### 2.8. Kleptomania

Little is known about the perception of patients with kleptomania. Regarding the disorders included in the current study, the desire for social distance ranked the highest among people with kleptomania in a study by Feldman and Crandall (2007), which indicates that people with this disorder might be perceived to be low in warmth within the SCM. There are no findings that would provide an indication for suggesting high or low ratings on the competence dimension. *We, therefore, hypothesize that kleptomania is perceived as low in warmth and moderate in competence within the SCM (H2h)*.

## 3. Materials and methods

### 3.1. Participants and design

We conducted an online study using a panel provider^1^ that recruited a sample representative for the German population in terms of age and gender (*N* = 297; 18–83 years of age, 52% female) for this study. An open-text item was used to assess participants’ current employment. The answers reflect a high degree of occupational and educational diversity of the sample: Retired 29.05%, in school, training, or university 7.09%, blue-collar worker 6.42%, white-collar worker 6.42%, without work and on parental leave 5.74%, freelancer 3.72%, specialized worker 3.38%, salesperson/distributor 3.38%, administration 2.70%, technician 2.36%, teacher 2.03%, nursing/medical staff 2.03%, consultant 1.69%, pedagogue 1.69%, CEO/owner 1.35%, engineer 1.35%, and many more. 3.7% of the sample (11 participants) stated that they themselves were affected by the disorder they assessed (5 with MDD, 2 with ADD and 1 with phobia, anorexia and kleptomania respectively). 42.74% of the sample (127 participants) stated that they know someone with the respective disorder in their social environment. 53.54% of the sample (159 participants) stated that neither themselves nor someone in their social environment was affected by the disorder. The participants reported an average personal contact with people with the respective disorders of 2.17 [SD = 1.11; “How do you rate your personal experience with people with (mental disorder)?”; 5-point Likert scale ranging from 1: very little to 5: a lot of contact] and an average expert knowledge about the disorders of 2.16 [SD = 1.08; “How do you rate your expert knowledge of people with (mental disorder)?”; 5-point Likert scale ranging from 1: very little to 5: high degree of expert knowledge]. The study was conceptualized as a between-subject design, in that participants answered questions concerning one of eight disorders (MDD, phobia, OCD, BPD, ADD, anorexia, schizophrenia and kleptomania). Following previous studies applying the SCM (e.g., Cuddy et al., 2004; Caprariello et al., 2009), we chose a between-subject design to avoid that participants make their judgments in comparison to the other included disorders (sensitivity effects). Furthermore, the between-subject design was chosen to minimize consistency effects (striving for a contradiction-free response), practice and fatigue effects, as well as demand characteristics (guessing the hypotheses) that are common to within-designs. Participants were randomly assigned to one condition/mental disorder, resulting in the following distribution: Schizophrenia 11%, MDD 16%, ADD 11%, OCD 16%, BPD 10%, phobia 11%, anorexia 12%, and kleptomania 13%.

### 3.2. Materials and procedure

After providing informed consent, participants indicated their gender, nationality, and age as well as whether they were from a city with a population of more than 100,000 inhabitants. This was followed by the warmth and competence ratings with regard to a randomly selected mental disorder. In line with Sadler et al. (2012), we presented groups as individuals with a certain disorder. We used a German short version (Asbrock, 2010) of the original warmth and competence measure by Fiske et al. (1999), which included three items for each dimension (warmth: likable, warm, good-natured; competence: competent, competitive, independent using a five-point Likert scale from 1: not at all to 5: completely). Asbrock (2010) reported excellent reliability scores for the short measure with Cronbach’s α of 0.86 for warmth and 0.98 for competence. The aggregated reliability scores in our sample are 0.72 for warmth and 0.74 for competence. For the different disorders, the scores for warmth vary between 0.52 (anorexia) and 0.78 (BPD), whereas the scores for the competence dimension vary between 0.51 (BDP) and 0.82 (schizophrenia). After the warmth and competence ratings, personal contact and expert knowledge were assessed as described above. At the end of the study and for exploratory purposes, participants answered questions about their training and occupation, as well as one question concerning data quality. The respective results are not reported here but can be provided upon request. Participants received a fixed payment based on the regulations of the online panel provider.

To test hypothesis 1, which claims distinct patterns for different mental disorders for warmth and competence, we conducted a Kruskal-Wallis Test and a hierarchical cluster analysis (Ward’s method). In order to test the disorder specific hypotheses H2a-h, we conducted one *t*-test for each dimension against the test value 3 (middle of the used Likert scale). In cases where we hypothesized high or low ratings, we ran one-tailed *t*-tests due to directional hypotheses, when we hypothesized moderate ratings, we ran two-tailed *t*-tests. We partly hypothesized moderate ratings on warmth and competence dimensions, which statistically equal null hypotheses. Therefore, we additionally run Bayesian *t*-tests (Rouder et al., 2009) for all non-significant *t*-tests as described in the “Bayes Factor” package (Morey et al., 2015) in R. Within this method, the Bayes Factor will indicate the probability of the null (moderate values, BF > 1) or alternative hypothesis (high/low values, BF < 1) given the observational data. Bayesian *t*-tests were calculated online^2^. We also report warmth and competence scores for the entire sample and overall disorders to assess whether the overarching category of people with mental disorders is consistent with the results of previous studies.

## 4. Results

**Figure 1:**
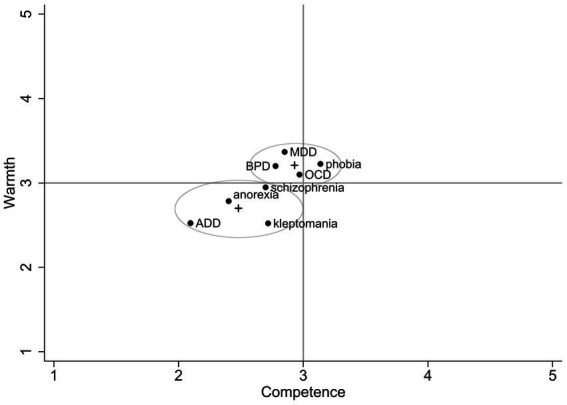
Two cluster-solution of different mental disorders within the SCM of the general public.

Descriptively, the different mental disorders appear to vary on the two dimensions (see Figure 1; see also Table 1 for mean values and standard deviations).

**Table 1:** Means and standard deviations of warmth and competence ratings for disorders in the general public.

Disorder	Warmth *M* (SD)	Competence *M* (SD)
Schizophrenia (*N* = 32)	2.95 (0.46)	2.70 (0.80)
ADD (*N* = 35)	2.52 (0.63)	2.10 (0.63)
MDD (*N* = 49)	3.37 (0.59)	2.85 (0.77)
BPD (*N* = 30)	3.20 (0.65)	2.78 (0.54)
Anorexia (*N* = 34)	2.78 (0.49)	2.40 (0.65)
Specific phobia (*N* = 34)	3.23 (0.45)	3.14 (0.71)
OCD (*N* = 44)	3.10 (0.59)	2.97 (0.64)
Kleptomania (*N* = 39)	2.52 (0.71)	2.72 (0.85)
Total (*N* = 297)	2.97 (0.65)	2.72 (0.76)

This is statistically confirmed by a Kruskal-Wallis Test, indicating that individuals have distinct stereotypes for different mental disorders with regard to warmth (*χ*^2^(7) = 66.02, *p* < 0.001) and competence *χ*^2^(7) = 45.08, *p* < 0.001). The hierarchical cluster analysis (Ward’s method, minimizing within-cluster variance and maximizing between-cluster variance), reveals a two-cluster solution (see Figure 1). K-means cluster analysis with parallel threshold method assigned each group to a cluster. The first cluster is named the moderate warmth and moderate competence cluster (MW/MC) and includes the disorders MDD, BPD, phobia, and OCD with cluster means of 3.22 for warmth and 2.93 for competence. The second cluster is named the low warmth and low competence cluster (LW/LC), which includes the disorders anorexia, ADD, kleptomania, and schizophrenia with cluster means of 2.69 for warmth and 2.48 for competence. The cluster means differ significantly regarding warmth (*t* = −7.93, *p* < 0.001) and competence (*t* = −5.31, *p* < 0.001). One-sample *t*-tests reveal mixed results concerning hypotheses 2a-h, with three fully supported hypotheses, four partly supported hypotheses, and one rejected hypothesis (see Table 2). We also calculated the averages for the SCM dimensions across all mental disorders, revealing moderate ratings for warmth (*M* = 2.97, SD = 0.65) and slightly lower ratings for competence (*M* = 2.72, SD = 0.76).

**Table 2:** One-sample *t*-tests against the middle of the scale of 3 and Bayes factor for non-significant *t*-tests.

Disorder	Warmth *t*	Competence *t*	Warmth Bayes factor	Competence Bayes factor	Hypotheses
Schizophrenia (*N* = 32)	−0.65	−2.14*	4.37		2a: partly supported
ADD (*N* = 35)	−4.49***	−8.44***			2b: supported
MDD (*N* = 49)	4.39***	−1.36		2.71	2c: partly supported
BPD (*N* = 30)	1.69	−2.25*	1.44		2d: not supported
Anorexia (*N* = 34)	−2.56**	−5.36***			2e: partly supported
Specific phobia (*N* = 34)	2.89**	1.13		3.02	2f: supported
OCD (*N* = 44)	1.11	−0.31	3.46	5.85	2 g: supported
Kleptomania (*N* = 39)	−4.22***	−2.07*			2 h: partly supported

*p < 0.05, **p < 0.01, ***p < 0.001. All results with moderate ratings, which revealed no significant results within one-sample *t*-tests, were confirmed by Bayesian *t*-tests.

Exploratory multiple regression analyses predicting warmth or competence by participant gender, age, and personal contact reveal that women gave significantly higher warmth ratings (*b* = 0.250, *p* = 0.001) but do not differ from male participants concerning competence ratings (*b* = 0.092, *p* = 0.325). Age is a significant predictor for perceived competence (*b* = −0.006, *p* = 0.042), but not for warmth (*b* = −0.003, *p* = 0.209). Furthermore, the degree of personal contact in our overall sample is a predictor for warmth (*b* = 0.129, *p* < 0.001) but not competence ratings (*b* = 0.008, *p* = 0.847). Based on these results, we rerun the main analyses separately for male vs. female participants, older vs. younger participants, and participants with high vs. low personal contact (the last two *via* median split): All conclusions from the above-mentioned Kruskal-Wallis Tests remain unchanged when doing so. The results listed in Table 2 partly deviate for different subgroups (e.g., one-sample *t*-tests regarding warmth are no longer significant within the subgroups MDD and specific phobia in male and anorexia in female participants) but the findings should not be overinterpreted since these subgroup analyses reduce statistical power.

## 5. Discussion

Distinct patterns of warmth and competence stereotypes for eight different mental disorders were observed (whereas some disorders are located in different clusters than expected). Thus, in line with Sadler et al. (2012) and the more recent contribution of Sönmez and Karaoğlu (2022) including Turkish undergraduates, results indicate that people with mental disorders are not perceived as one indefinable social group but rather that the perception does differentiate between different disorders. The general public reported negative stereotypes for people with ADD, anorexia, and kleptomania with regard to warmth and competence, whereas people with schizophrenia and BDP are only negatively rated with regard to competence. The stereotypical perception of people with MDD and phobia is positive on the warmth dimension. Averaging all groups revealed moderate ratings for warmth and somewhat lower ratings for competence in a German sample, which is in line with previous results based on stereotypical perceptions of the overarching category of “people with mental disorders/illness” (Asbrock, 2010; Sadler et al., 2012).

Based on theoretical accounts and previous empirical results, disorder specific hypotheses regarding the perceived warmth and competence of different mental disorders were derived for this project. These predictions were supported by four out of eight disorders (ADD, MDD, specific Phobia, and OCD). For the other disorders, hypotheses were not (BPD) or only partially (schizophrenia, anorexia, kleptomania) supported, indicating a potential involvement of other factors such as cultural differences (since most studies have been conducted in the US) or other unknown determinants.

Some of the disorder specific results can be related to other research. For example, according to MDD, the findings are mixed: Görzig et al. (2019) as well as Follmer and Jones (2017) reported that people with depression were rated low in warmth and competence, whereas in Sadler et al. (2015) they were rated high in warmth and low in competence. In our study in turn they were rated as high in warmth and moderate in competence. Schizophrenia was located in the low warmth/low competence cluster and the competence rating of this disorder significantly deviated from the midpoint of the scale. However, regarding perceived warmth, we did not find a significant deviation from the scale mean. This finding somewhat contradicts other results showing that people with schizophrenia were rated as being low in both dimensions (Sadler et al., 2012; Sönmez and Karaoğlu, 2022). Also, other findings deviate from what has been found by other researchers: For example, the competence scores for anorexia and BPD have been low relative to the other disorders, which deviates from the findings of Sadler et al. (2012) and Sönmez and Karaoğlu (2022). However, it should be noted that these studies assessed perceptions of “eating disorders” in general and not of “anorexia” specifically.

Other studies on stereotypic perceptions of people with mental disorders differ from our study with regard to several aspects that could potentially account for the divergent results: Our study was conducted in Germany. Therefore, cultural differences might contribute to different results in other countries such as the US (Sadler et al., 2012) and Turkey (Sönmez and Karaoğlu, 2022). We used a sample representative of the German population in terms of age and gender and with a higher diversity than other studies, which could also lead to different results. Also, deviating from other studies, we asked about personal perceptions rather than assumed opinions in society, possibly resulting in a higher degree of social desirability and therefore in more positive ratings (Kotzur et al., 2020). Given the mixed findings, future studies could readdress the perception of people with mental disorders using different measures and contexts.

Boysen (2017) included the concept of internalizing vs. externalizing mental disorders in his work about the stereotypical perception of people with mental disorders within the SCM. Internalizing disorders share the common feature that distress is processed more inwardly on a social, behavioral, and emotional level (Krueger et al., 2001; Achenbach et al., 2016). Externalizing disorders, on the other hand, tend to transfer distress outwards, which manifests as observable behaviors, such as impulsive, aggressive, disruptive, or addiction-related behaviors. Similar to Boysen (2017), we found that more externalizing disorders (i.e., ADD, kleptomania, and schizophrenia) are associated with less perceived competence and warmth, whereas more internalizing disorders (i.e., MDD, specific Phobia, OCD) are perceived more positively on both dimensions. It appears plausible that people with more externalizing as compared to internalizing disorders are located in the LW/LC cluster, because they might appear impulsive, unpredictable, and potentially dangerous (i.e., lack of perceived warmth). The clusters retrieved in our studies are also comparable to those reported in Sadler et al. (2012), in that five out of six (except anorexia) included similar disorders and were located at similar positions in the two-dimensional space.

In line with previous research, women gave more positive ratings (Yuan et al., 2016). Specifically, they perceived people with mental disorders as higher in warmth. No gender differences were found for perceived competence. Again, in line with Yuan et al. (2016) we found a negative relationship between age and the stereotypical perception, here regarding competence ratings (but see also Angermeyer and Dietrich, 2006). Thus, future research should include demographically diverse samples considering potential influences of demographic variables such as participant age and gender.

In sum, our data indicates that the stereotypical perception of eight different mental disorders [major depressive disorder (MDD), specific phobia, obsessive compulsive disorder (OCD), emotional-unstable personality disorder, borderline type (BPD), alcohol dependence disorder (ADD), anorexia nervosa, schizophrenia, and kleptomania] can be displayed on the two dimensions of warmth and competence of the Stereotype Content Model (SCM) in Germany. Aggregating the values of all disorders resulted in moderate warmth ratings and lower competence ratings, which is consistent with prior research examining the category “people with mental disorders” within the SCM (Sadler et al., 2012).

### 5.1. Limitations

As a limitation of the study, it must be mentioned that some of the reliability scores for the warmth and competence ratings were questionable (below 0.70), indicating that the participants rated the same mental disorder very differently on the two dimensions. Low reliabilities for some groups have been also reported in other studies (e.g., Nett et al., 2020; Chen et al., 2021). Thus, it is possible that some groups cannot be exclusively or sufficiently described by two dimensions. Future research on the stereotypical perception of mental disorders might thus consider alternative measures (e.g., the ABC-Model by Koch et al., 2016). Deviating from other research in this research area (e.g., Sadler et al., 2012), we asked for participants’ personal perceptions of the different groups [“How likeable do you think people with (mental illness) are?”], rather than the assumed perceptions of the general population [“As viewed by society, how likeable are people with (mental illness)?”]. Doing so allows us to compare the perception across different samples. However, this decision might have implications for the interpretation of the data. Kotzur et al. (2020) for example have shown that questions about personal perceptions within SCM result in more positive ratings than questions about the society’s perceptions, presumably for reasons of social desirability. Thus, providing our participants with the alternative measure might have resulted in even more negative ratings. Another limitation of the study is that the data collection already took place in 2016. However, since prejudices and social stigmas change only slowly (Möller-Leimkühler, 2004), we assume that the data is still relevant. In addition, this is a study with an explicit measurement of stereotypes that may cause effects of social desirability. Gonzalez-Sanguino et al. (2019) indicated that the use of implicit measurement methods of attitudes may be advantageous in future studies. With regard to the here applied between-subject design, one has to consider a recently published comprehensive reanalysis of published studies including the warmth and competence dimensions that has shown that studies using a within-subject design overall reached a better model fit compared to studies with a between-subject design (Friehs et al., 2022), leading us the conclusion that we would use within-subject designs in future SCM studies.

### 5.2. Conclusion and future directions

It is important to understand how people with mental disorders are perceived by the general public – because these perceptions can lead to predictions of specific emotional and behavioral tendencies (Cuddy et al., 2007). Although there already exists a considerable amount of research concerning discrimination of people with mental disorders in social or occupational life, many lack a proper theoretical framework or use an experimental design with insufficient internal validity. The SCM can offer an adequate theoretical framework to investigate such stereotypes toward people with different mental disorders. Future research can complement our research by employing the data driven ABC-Model by Koch et al. (2016), where the dimensions A – agency and C – communion are related to competence and warmth and are complemented by a third dimension B – belief, emphasizing the role of traditional vs. progressive tendencies for social judgments. The present research provides data on mapped stereotypes toward people with specific mental disorders from a representative sample in Germany in addition to the existing literature. A future cross-national or cross-cultural perspective on stereotypes of people with mental disorders within the SCM (and ABC) could be investigated, as existing research suggests that there are, e.g., differences between Western and Non-western cultures with regard to affective, cognitive, and behavioral dimensions of stigma (Ahmed et al., 2020). Future research could also investigate further predictors of stereotypical perceptions. Our explorative analyses suggest that the frequency of personal contact with people with mental disorders might have a positive influence on the stereotypical perception of warmth. However, previous research results in this respect are mixed: Pettigrew and Tropp (2000) found more positive stereotypical perceptions of people with mental disorders with increasing contact. Other studies found that healthcare professionals, who typically have frequent personal contact, perceived people with mental disorders more negatively than the general public (for a review, see Alshahrani, 2018). The findings by Kotzur and Wagner (2021) and Kotzur et al. (2019) suggest that the frequency of positive or negative experiences determines stereotype perceptions. Thus, it would be of interest to investigate the extent to which stereotypical perceptions (within the SCM framework) of people with mental disorders are influenced by positive and negative personal contact. Future studies could also take an intersectional perspective and consider some of the multiple variables already identified as relevant to stereotype perception (Fiske, 2010), such as gender, in assessing perceptions of people with mental disorders. For example, one could examine whether stereotype perceptions are different for men or women with certain mental disorders since there is evidence that certain disorders are associated with masculinity or femininity, affecting social perception (Boysen, 2017). From a practical perspective, our results suggest that specific therapy and awareness programs are crucial, since people with different disorders are perceived differently by the general public. In line with previous findings (Sadler et al., 2012), our results for example show that people with ADD are perceived more negatively than people with MDD or phobia. Based on these results, one can recommend developing specific awareness programs for the particularly negatively perceived disorders (e.g., ADD, schizophrenia, kleptomania), which might influence their recovery and reintegration process into society.

## Data availability statement

The datasets presented in this study can be found in online repositories. The names of the repository/repositories and accession number (s) can be found at: The materials and data can be found at https://osf.io/ukzn2/.

## Ethics statement

Ethical review and approval was not required for the study on human participants in accordance with the local legislation and institutional requirements. The patients/participants provided their written informed consent to participate in this study.

## Author contributions

AD and CS developed the study design and collected the data. RA conducted the literature research, analyzed data, and documented the results together with AD. RA wrote the article which was revised by AD. All authors contributed to the article and approved the submitted version.

## Funding

Funding for data collection via the panel provider https://www.consumerfieldwork.com/ was provided by resources of the University of Siegen.

## Conflict of interest

The authors declare that the research was conducted in the absence of any commercial or financial relationships that could be construed as a potential conflict of interest.

## Publisher’s note

All claims expressed in this article are solely those of the authors and do not necessarily represent those of their affiliated organizations, or those of the publisher, the editors and the reviewers. Any product that may be evaluated in this article, or claim that may be made by its manufacturer, is not guaranteed or endorsed by the publisher.

## Footnotes

^1^ https://www.consumerfieldwork.com/

^2^ Bayesian t-test calculator provided by Norouzian et al. (2018) produces the same results and can be found here: https://rezanorouzian.shinyapps.io/bayes-t/

## Abbreviations

SCM: Stereotype Content Model
WHO: World Health Organization
ICD: International Classification of Diseases
MDD: major depressive disorder
OCD: obsessive compulsive disorder
BPD: borderline personality disorder/emotional-unstable personality disorder, borderline type
ADD: alcohol dependence disorder
MW/MC-cluster: moderate warmth and moderate competence cluster
MW/MC-cluster: low warmth and low competence cluster

## References

[ref1] Achenbach T. M. Ivanova M. Y. Rescorla L. A. Turner L. V. Althoff R. R. (2016). Internalizing/externalizing problems: review and recommendations for clinical and research applications. J. Am. Acad. Child Adolesc. Psychiatry 55, 647–656. doi: 10.1016/j.jaac.2016.05.012, PMID: 27453078

[ref2] Ahmed S. Birtel M. D. Pyle M. Morrison A. P. (2020). Stigma towards psychosis: cross-cultural differences in prejudice, stereotypes, and discrimination in White British and south Asians. J. Community Appl. Soc. Psychol. 30, 199–213. doi: 10.1002/casp.2437

[ref4] Alonso J. Angermeyer M. C. Bernert S. Bruffaerts R. Brugha T. S. Bryson H.. (2004). Disability and quality of life impact of mental disorders in Europe: results from the European study of the epidemiology of mental disorders (ESEMeD) project. Acta Psychiatr. Scand. 109, 38–46. doi: 10.1111/j.1600-0047.2004.00329.x, PMID: 15128386

[ref5] Alshahrani W. (2018). A literature review of healthcare professionals’ attitudes towards patients with mental illness. J. Med. Res. Health Educ. 2, 1–5.

[ref6] Angermeyer M. C. Dietrich S. (2006). Public beliefs about and attitudes towards people with mental illness: a review of population studies. Acta Psychiatr. Scand. 113, 163–179. doi: 10.1111/j.1600-0447.2005.00699.x, PMID: 16466402

[ref7] Angermeyer M. C. Matschinger H. (2003). Public beliefs about schizophrenia and depression: similarities and differences. Soc. Psychiatry Psychiatr. Epidemiol. 38, 526–534. doi: 10.1007/s00127-003-0676-6, PMID: 14504738

[ref8] Angermeyer M. C. Matschinger H. (2004). The stereotype of schizophrenia and its impact on discrimination against people with schizophrenia: results from a representative survey in Germany. Schizophr. Bull. 30, 1049–1061. doi: 10.1093/oxfordjournals.schbul.a007120, PMID: 15954207

[ref9] Angermeyer M. C. Matschinger H. (2005). Causal beliefs and attitudes to people with schizophrenia. Trend analysis based on data from two population surveys in Germany. Br. J. Psychiatry 186, 331–334. doi: 10.1192/bjp.186.4.331 15802691

[ref10] Angermeyer M. C. Matschinger H. Schomerus G. (2013). Attitudes towards psychiatric treatment and people with mental illness: changes over two decades. Br. J. Psychiatry 203, 146–151. doi: 10.1192/bjp.bp.112.122978, PMID: 23787060

[ref11] Asbrock F. (2010). Stereotypes of social groups in Germany in terms of warmth and competence. Soc. Psychol. 41, 76–81. doi: 10.1027/1864-9335/a000011

[ref12] Ashmore R. D. Del Boca E. K. (1981). “Conceptual approaches to stereotypes and stereotyping” in Cognitive Processes in Stereotyping and Intergroup Behavior. ed. Hamilton D. L. (London: Psychology Press), 1–35.

[ref13] Asselmann E. Beesdo-Baum K. Hamm A. Schmidt C. O. Hertel J. Grabe H. J.. (2019). Lifetime and 12-month prevalence estimates for mental disorders in northeastern Germany: findings from the study of health in Pomerania. Eur. Arch. Psychiatry Clin. Neurosci. 269, 341–350. doi: 10.1007/s00406-018-0911-5, PMID: 29948253

[ref15] Boysen G. A. (2017). Exploring the relation between masculinity and mental illness stigma using the stereotype content model and BIAS map. J. Soc. Psychol. 157, 98–113. doi: 10.1080/00224545.2016.1181600, PMID: 27110638

[ref16] Caprariello P. A. Cuddy A. J. Fiske S. T. (2009). Social structure shapes cultural stereotypes and emotions: a causal test of the stereotype content model. Group Process. Intergroup Relat. 12, 147–155. doi: 10.1177/1368430208101053, PMID: 24285928 PMC3839230

[ref17] Chen V. H. H. Ahmed S. Chib A. (2021). The role of social media behaviors and structural intergroup relations on immigrant stereotypes. Int. J. Commun. 15, 4085–4109. doi: 1932–8036/20210005

[ref18] Clement S. Schauman O. Graham T. Maggioni F. Evans-Lacko S. Bezborodovs N.. (2015). What is the impact of mental health-related stigma on help-seeking? A systematic review of quantitative and qualitative studies. Psychol. Med. 45, 11–27. doi: 10.1017/S0033291714000129, PMID: 24569086

[ref19] Corrigan P. (2004). How stigma interferes with mental health care. Am. Psychol. 59, 614–625. doi: 10.1037/0003-066X.59.7.614 15491256

[ref21] Crisafulli M. A. Von Holle A. Bulik C. M. (2008). Attitudes towards anorexia nervosa: the impact of framing on blame and stigma. Int. J. Eat. Disord. 41, 333–339. doi: 10.1002/eat.20507, PMID: 18186057

[ref22] Cuddy A. J. Fiske S. T. Glick P. (2004). When professionals become mothers, warmth doesn't cut the ice. J. Soc. Issues 60, 701–718. doi: 10.1111/j.0022-4537.2004.00381.x

[ref23] Cuddy A. J. Fiske S. T. Glick P. (2007). The BIAS map: behaviors from intergroup affect and stereotypes. J. Pers. Soc. Psychol. 92, 631–648. doi: 10.1037/0022-3514.92.4.631, PMID: 17469949

[ref24] Cuddy A. J. Fiske S. T. Glick P. (2008). Warmth and competence as universal dimensions of social perception: the stereotype content model and the BIAS map. Adv. Exp. Soc. Psychol. 40, 61–149. doi: 10.1016/S0065-2601(07)00002-0

[ref25] Cuddy A. J. Fiske S. T. Kwan V. S. Glick P. Demoulin S. Leyens J. P.. (2009). Stereotype content model across cultures: towards universal similarities and some differences. Br. J. Soc. Psychol. 48, 1–33. doi: 10.1348/014466608X314935, PMID: 19178758 PMC3912751

[ref26] Curcio C. Corboy D. (2020). Stigma and anxiety disorders: a systematic review. Stigma Health 5, 125–137. doi: 10.1037/sah0000183

[ref27] Deans C. Meocevic E. (2006). Attitudes of registered psychiatric nurses towards patients diagnosed with borderline personality disorder. Contemp. Nurse 21, 43–49. doi: 10.5172/conu.2006.21.1.43, PMID: 16594881

[ref29] Durante F. Fiske S. T. Gelfand M. J. Crippa F. Suttora C. Stillwell A.. (2017). Ambivalent stereotypes link to peace, conflict, and inequality across 38 nations. Proc. Natl. Acad. Sci. 114, 669–674. doi: 10.1073/pnas.1611874114, PMID: 28069955 PMC5278477

[ref30] Durante F. Fiske S. T. Kervyn N. Cuddy A. J. Akande A. Adetoun B. E.. (2013). Nations' income inequality predicts ambivalence in stereotype content: how societies mind the gap. Br. J. Soc. Psychol. 52, 726–746. doi: 10.1073/pnas.161187411, PMID: 23039178 PMC3855559

[ref31] Ebneter D. S. Latner J. D. O’Brien K. S. (2011). Just world beliefs, causal beliefs, and acquaintance: associations with stigma toward eating disorders and obesity. Personal. Individ. Differ. 51, 618–622. doi: 10.1016/j.paid.2011.05.029

[ref32] Faul F. Erdfelder E. Lang A.-G. Buchner A. (2007). G*power 3: a flexible statistical power analysis program for the social, behavioral, and biomedical sciences. Behav. Res. Methods 39, 175–191. doi: 10.3758/BF03193146, PMID: 17695343

[ref33] Feldman D. B. Crandall C. S. (2007). Dimensions of mental illness stigma: what about mental illness causes social rejection? J. Soc. Clin. Psychol. 26, 137–154. doi: 10.1521/jscp.2007.26.2.137

[ref34] Fiske S. T. (1999). “Stereotyping, prejudice, and discrimination” in Handbook of Social Psychology. eds. Gilbert D. T. Fiske S. T. Lindzey G., vol. 55. 4th ed (New York, NY: McGraw-Hill), 473–489.

[ref35] Fiske S. T. (2010). Venus and Mars or down to earth: stereotypes and realities of gender differences. Perspect. Psychol. Sci. 5, 688–692. doi: 10.1177/1745691610388768, PMID: 23678365 PMC3652639

[ref36] Fiske S. T. (2012). Warmth and competence: stereotype content issues for clinicians and researchers. Can. Psychol. 53, 14–20. doi: 10.1037/a0026054, PMID: 24155504 PMC3801417

[ref37] Fiske S. T. (2018). Stereotype content: warmth and competence endure. Curr. Dir. Psychol. Sci. 27, 67–73. doi: 10.1177/0963721417738825, PMID: 29755213 PMC5945217

[ref38] Fiske S. T. Cuddy A. J. Glick P. Xu J. (2002). A model of (often mixed) stereotype content: competence and warmth respectively follow from perceived status and competition. J. Pers. Soc. Psychol. 82, 878–902. doi: 10.1037/0022-3514.82.6.878, PMID: 12051578

[ref39] Fiske S. T. Durante F. (2016). “Stereotype content across cultures: variations on a few themes” in Handbook of Advances in Culture and Psychology. eds. Gelfand M. J. Chiu C.-Y. Hong Y.-Y. (Oxford: Oxford University Press), 209–258.

[ref40] Fiske S. T. Xu J. Cuddy A. C. Glick P. (1999). (dis) respecting versus (dis) liking: status and interdependence predict ambivalent stereotypes of competence and warmth. J. Soc. Issues 55, 473–489. doi: 10.1111/0022-4537.00128

[ref41] Follmer K. B. Jones K. S. (2017). Stereotype content and social distancing from employees with mental illness: the moderating roles of gender and social dominance orientation. J. Appl. Soc. Psychol. 47, 492–504. doi: 10.1111/jasp.12455

[ref42] Friehs M.-T. Kotzur P. F. Böttcher J. Zöller A.-K. C. Lüttmer T. Wagner U.. (2022). Examining the structural validity of stereotype content scales – a preregistered re-analysis of published data and discussion of possible future directions. Int. Rev. Soc. Psychol. 35, 1–18. doi: 10.5334/irsp.613

[ref43] Gärtner L. Asbrock F. Euteneuer F. Rief W. Salzmann S. (2022). Self-stigma among people with mental health problems in terms of warmth and competence. Front. Psychol. 13:877491. doi: 10.3389/fpsyg.2022.877491, PMID: 35774956 PMC9237425

[ref44] Gonzalez-Sanguino C. Munoz M. Castellanos M. A. Perez-Santos E. Orihuela-Villameriel T. (2019). Study of the relationship between implicit and explicit stigmas associated with mental illness. Psychiatry Res. 272, 663–668. doi: 10.1016/j.psychres.2018.12.172, PMID: 30616138

[ref45] Görzig A. Bedrosova M. Machackova H. (2019). Do stereotypes of mental and developmental disorders predict bystander intentions in cyberbullying? An application of the stereotype content model. Int. J. Dev. Sci. 13, 83–95. doi: 10.3233/DEV-190270

[ref46] Grausgruber A. Schöny W. Grausgruber-Berner R. Koren G. Apor B. F. Wancata J.. (2009). Schizophrenia has many faces - evaluation of the Austrian anti-stigma-campaign 2000-2002. Psychiatr. Prax. 36, 327–333. doi: 10.1055/s-0029-1220386, PMID: 19724998

[ref47] Griffiths K. M. Christensen H. Jorm A. F. (2008). Predictors of depression stigma. BMC Psychiatry 8, 1–12. doi: 10.1186/1471-244X-8-25, PMID: 18423003 PMC2386456

[ref48] Henderson C. Robinson E. Evans-Lacko S. Thornicroft G. (2017). Relationships between anti-stigma programme awareness, disclosure comfort and intended help-seeking regarding a mental health problem. Br. J. Psychiatry 211, 316–322. doi: 10.1192/bjp.bp.116.195867, PMID: 28935661 PMC5663972

[ref49] Hinshaw S. P. Stier A. (2008). Stigma as related to mental disorders. Annu. Rev. Clin. Psychol. 4, 367–393. doi: 10.1146/annurev.clinpsy.4.022007.141245 17716044

[ref50] Hipes C. Lucas J. Phelan J. C. White R. C. (2016). The stigma of mental illness in the labor market. Soc. Sci. Res. 56, 16–25. doi: 10.1016/j.ssresearch.2015.12.001 26857169

[ref51] Holzinger A. Floris F. Schomerus G. Carta M. G. Angermeyer M. C. (2012). Gender differences in public beliefs and attitudes about mental disorder in western countries: a systematic review of population studies. Epidemiol. Psychiatr. Sci. 21, 73–85. doi: 10.1017/S2045796011000552, PMID: 22670415

[ref52] Kanahara S. (2006). A review of the definitions of stereotype and a proposal for a progressional model. Individ. Differ. Res. 4, 306–321.

[ref53] Koch A. Imhoff R. Dotsch R. Alves H. Unkelbach C. (2016). The ABC of stereotypes about groups: agency/socioeconomic success, conservative-progressive beliefs, and communion. J. Pers. Soc. Psychol. 110, 675–709. doi: 10.1037/pspa0000046, PMID: 27176773

[ref54] Kotzur P. F. Schäfer S. J. Wagner U. (2019). Meeting a nice asylum seeker: intergroup contact changes stereotype content perceptions and associated emotional prejudices, and encourages solidarity-based collective action intentions. Br. J. Soc. Psychol. 58, 668–690. doi: 10.1111/bjso.12304, PMID: 30512181

[ref55] Kotzur P. F. Veit S. Namyslo A. Holthausen M. A. Wagner U. Yemane R. (2020). ‘Society thinks they are cold and/or incompetent, but I do not’: stereotype content ratings depend on instructions and the social group's location in the stereotype content space. Br. J. Soc. Psychol. 59, 1018–1042. doi: 10.1111/bjso.12375, PMID: 32212336

[ref56] Kotzur P. F. Wagner U. (2021). The dynamic relationship between contact opportunities, positive and negative intergroup contact, and prejudice: a longitudinal investigation. J. Pers. Soc. Psychol. 120, 418–442. doi: 10.1037/pspi0000258, PMID: 32700961

[ref57] Krueger R. F. McGue M. Iacono W. G. (2001). The higher-order structure of common DSM mental disorders: internalization, externalization, and their connections to personality. Personal. Individ. Differ. 30, 1245–1259. doi: 10.1016/S0191-8869(00)00106-9

[ref58] Levey S. Howells K. Levey S. (1995). Dangerousness, unpredictability and the fear of people with schizophrenia. J. Forensic Psychiatry 6, 19–39. doi: 10.1080/09585189508409874

[ref59] Link B. G. Phelan J. C. Bresnahan M. Stueve A. Pescosolido B. A. (1999). Public conceptions of mental illness: labels, causes, dangerousness, and social distance. Am. J. Public Health 89, 1328–1333. doi: 10.2105/ajph.89.9.1328, PMID: 10474548 PMC1508784

[ref60] Link B. G. Yang L. H. Phelan J. C. Collins P. Y. (2004). Measuring mental illness stigma. Schizophr. Bull. 30, 511–541. doi: 10.1093/oxfordjournals.schbul.a007098 15631243

[ref61] Macrae C. N. Bodenhausen G. V. (2000). Social cognition: thinking categorically about others. Annu. Rev. Psychol. 51, 93–120. doi: 10.1146/annurev.psych.51.1.93 10751966

[ref62] Mehta N. Kassam A. Leese M. Butler G. Thornicroft G. (2009). Public attitudes towards people with mental illness in England and Scotland, 1994–2003. Br. J. Psychiatry 194, 278–284. doi: 10.1192/bjp.bp.108.052654, PMID: 19252160

[ref63] Möller-Leimkühler A. M. (2004). Stigmatisierung psychisch Kranker aus der Perspektive sozialpsychologischer Stereotypenforschung [why are stereotypes about mentally ill so resistant? Lessons from social psychology]. Fortschr. Neurol. Psychiatr. 72, 36–44. doi: 10.1055/s-2003-812456, PMID: 14745688

[ref500] Morey R. D. Rouder J. N. Jamil T. Morey M. R. D. (2015). Package ‘bayesfactor’. Available at: https://cran.utstat.utoronto.ca/web/packages/BayesFactor/BayesFactor.pdf, PMID: 17695343

[ref64] Nett T. Dorrough A. Jekel M. Glöckner A. (2020). Perceived biological and social characteristics of a representative set of German first names. Soc. Psychol. 51, 17–34. doi: 10.1027/1864-9335/a000383

[ref300] Norouzian R. de Miranda M. Plonsky L. (2018). The Bayesian revolution in second language research: An applied approach. Language Learning. 68, 1032–1075. doi: 10.1111/lang.12310, PMID: 24285928

[ref65] Pescosolido B. A. Martin J. K. Long J. S. Medina T. R. Phelan J. C. Link B. G. (2010). “A disease like any other”? A decade of change in public reactions to schizophrenia, depression, and alcohol dependence. Am. J. Psychiatr. 167, 1321–1330. doi: 10.1176/appi.ajp.2010.09121743, PMID: 20843872 PMC4429867

[ref66] Pescosolido B. A. Monahan J. Link B. G. Stueve A. Kikuzawa S. (1999). The public's view of the competence, dangerousness, and need for legal coercion of persons with mental health problems. Am. J. Public Health 89, 1339–1345. doi: 10.2105/ajph.89.9.1339, PMID: 10474550 PMC1508769

[ref67] Pettigrew T. F. Tropp L. R. (2000). “Does intergroup contact reduce prejudice: recent meta-analytic findings” in Reducing Prejudice and Discrimination. ed. Oskamp S. (Mahwah, NJ: Lawrence Erlbaum Associates Publishers), 93–114.

[ref68] Phelan J. C. Link B. G. Stueve A. Pescosolido B. A. (2000). Public conceptions of mental illness in 1950 and 1996: what is mental illness and is it to be feared? J. Health Soc. Behav. 41, 188–207. doi: 10.2307/2676305

[ref69] Read J. Haslam N. Sayce L. Davies E. (2006). Prejudice and schizophrenia: a review of the ‘mental illness is an illness like any other’ approach. Acta Psychiatr. Scand. 114, 303–318. doi: 10.1111/j.1600-0447.2006.00824.x, PMID: 17022790

[ref70] Reavley N. J. Jorm A. F. (2011). Stigmatizing attitudes towards people with mental disorders: findings from an Australian National Survey of mental health literacy and stigma. Aust. N. Z. J. Psychiatry 45, 1086–1093. doi: 10.3109/00048674.2011.621061, PMID: 22023236

[ref71] Roehrig J. P. McLean C. P. (2010). A comparison of stigma toward eating disorders versus depression. Int. J. Eat. Disord. 43, 671–674. doi: 10.1002/eat.20760, PMID: 19816860

[ref72] Ross C. A. Goldner E. M. (2009). Stigma, negative attitudes and discrimination towards mental illness within the nursing profession: a review of the literature. J. Psychiatr. Ment. Health Nurs. 16, 558–567. doi: 10.1111/j.1365-2850.2009.01399.x, PMID: 19594679

[ref400] Rouder J. N. Speckman P. L. Sun D. Morey R. D. Iverson G. (2009). Bayesian t tests for accepting and rejecting the null hypothesis. Psychon. Bull. Rev. 16, 225–237. doi: 10.3758/PBR.16.2.225, PMID: 19293088

[ref73] Rüsch N. Angermeyer M. C. Corrigan P. W. (2005). Mental illness stigma: concepts, consequences, and initiatives to reduce stigma. Eur. Psychiatry 20, 529–539. doi: 10.1016/j.eurpsy.2005.04.004, PMID: 16171984

[ref74] Sadler M. S. Kaye K. E. Vaughn A. A. (2015). Competence and warmth stereotypes prompt mental illness stigma through emotions. J. Appl. Soc. Psychol. 45, 602–612. doi: 10.1111/jasp.12323

[ref75] Sadler M. S. Meagor E. L. Kaye K. E. (2012). Stereotypes of mental disorders differ in competence and warmth. Soc. Sci. Med. 74, 915–922. doi: 10.1016/j.socscimed.2011.12.019, PMID: 22321391

[ref76] Schmitt M. T. Branscombe N. R. Postmes T. Garcia A. (2014). The consequences of perceived discrimination for psychological well-being: a meta-analytic review. Psychol. Bull. 140, 921–948. doi: 10.1037/a0035754, PMID: 24547896

[ref77] Schomerus G. Schwahn C. Holzinger A. Corrigan P. W. Grabe H. J. Carta M. G.. (2012). Evolution of public attitudes about mental illness: a systematic review and meta-analysis. Acta Psychiatr. Scand. 125, 440–452. doi: 10.1111/j.1600-0447.2012.01826.x, PMID: 22242976

[ref78] Sönmez B. Karaoğlu K. M. (2022). Contents of stereotypes toward mental illness. Curr. Psychol. 1-10:693. doi: 10.1007/s12144-022-03693-9

[ref79] Thompson A. H. Stuart H. Bland R. C. Arboleda-Florez J. Warner R. Dickson R. A. (2002). Attitudes about schizophrenia from the pilot site of the WPA worldwide campaign against the stigma of schizophrenia. Soc. Psychiatry Psychiatr. Epidemiol. 37, 475–482. doi: 10.1007/s00127-002-0583-2, PMID: 12242626

[ref80] World Health Organization [WHO]. (1993). The ICD-10 Classification of Mental and Behavioral Disorders. Geneva: World Health Organization.

[ref81] Yoshimura Y. Bakolis I. Henderson C. (2018). Psychiatric diagnosis and other predictors of experienced and anticipated workplace discrimination and concealment of mental illness among mental health service users in England. Soc. Psychiatry Psychiatr. Epidemiol. 53, 1099–1109. doi: 10.1007/s00127-018-1561-7, PMID: 29987387

[ref82] Yuan Q. Abdin E. Picco L. Vaingankar J. A. Shahwan S. Jeyagurunathan A.. (2016). Attitudes to mental illness and its demographic correlates among general population in Singapore. PLoS One 11:e0167297. doi: 10.1371/journal.pone.0167297, PMID: 27893796 PMC5125689

